# Isolation policies in musculoskeletal infection care: time to move from tradition to evidence

**DOI:** 10.5194/jbji-11-489-2026

**Published:** 2026-08-07

**Authors:** Laura Bessems, Jolien Onsea, Marjan Wouthuyzen-Bakker, Irene K. Sigmund, Tristan Ferry, Richard Kuehl, Martin Clauss, Alex Soriano, Ricardo Sousa, Annette Schuermans, Willem-Jan Metsemakers

**Affiliations:** 1 Department of Development and Regeneration, KU Leuven, 3000 Leuven, Belgium; 2 Department of Trauma Surgery, University Hospitals Leuven, 3000 Leuven, Belgium; 3 Department of Medical Microbiology and Infection Prevention, University Medical Center Groningen, Groningen, the Netherlands; 4 University of Groningen, 9713 GZ Groningen, the Netherlands; 5 Department of Orthopaedics and Traumatology, Medical University of Vienna, 1090 Vienna, Austria; 6 Infectious and Tropical Diseases Unit, Croix-Rousse Hospital, Hospices Civils de Lyon, 69004 Lyon, France; 7 Division of Infectious Diseases & Hospital Epidemiology, University Hospital Basel, 4031 Basel, Switzerland; 8 Department of Orthopaedics and Trauma Surgery, Center for Musculoskeletal Infections (ZMSI), University Hospital Basel, 4031 Basel, Switzerland; 9 Hospital Clinic of Barcelona, 08036 Barcelona, Spain; 10 IDIBAPS, 08036 Barcelona, Spain; 11 CIBERNIF, CIBER in Infectious Diseases, ISCIII, 28029 Madrid, Spain; 12 Orthopaedic Department, Porto Bone Infection Group (GRIP), ULS Santo António, 4099-001 Porto, Portugal; 13 Department of Infection Control and Epidemiology, University Hospitals Leuven, 3000 Leuven, Belgium; 14 Department of Public Health and Primary Care, KU Leuven – University of Leuven, 3000 Leuven, Belgium

## Abstract

A recent scoping review found no evidence supporting routine ward-level isolation or dedicated septic wards for non-multidrug-resistant organism musculoskeletal infections when standard precautions are applied, whereas targeted measures remain appropriate for multidrug-resistant organisms. An international survey revealed wide variation in attitudes and a persistent belief in supporting evidence, highlighting a gap between evidence and perception and supporting a differentiated, risk-based approach to isolation.

## 1 Viewpoint

Patient isolation has long been a central strategy in infection control. In orthopaedic practice, this traditionally involved dedicated septic wards or structural separation of infected patients from elective or trauma populations, reflecting concerns about cross-contamination in the presence of lifelong implants and limited treatment options (Kempf et al., 1985).

With the introduction of antibiotics and the widespread implementation of standard precautions, infection prevention strategies fundamentally changed. By the late twentieth century, the emphasis had shifted from architectural separation toward universally applied measures such as hand hygiene, environmental cleaning, and appropriate use of personal protective equipment, integrated into routine patient care (Prevention CDC, 2007; Haynes and Khardori, 2013).

In current healthcare practice, isolation is largely limited to patients with multidrug-resistant or highly transmissible organisms (MDROs), such as methicillin-resistant *Staphylococcus aureus* (MRSA). For most non-MDRO musculoskeletal infections (MSIs), routine isolation or patient cohorting is being increasingly questioned. Despite major advances in surgical techniques, antimicrobial therapy, and infection prevention, isolation policies in orthopaedic wards often remain driven by historical practice rather than available evidence.

This issue has become particularly relevant in the post-COVID-19 era, where constraints in bed capacity, single-room availability, and staffing have forced healthcare systems to reconsider long-standing practices. In this context, isolation is not only an infection control measure but also a determinant of care efficiency and resource allocation.

Therefore, a critical re-evaluation of classic isolation policies for patients with MSIs is warranted. Expanding on an earlier scoping review (Bessems et al., 2025), this article uses survey results to highlight the discrepancy between existing evidence and current isolation practices in MSI care, encouraging the adoption of a more risk-based approach.

## 2 Limited evidence supporting routine isolation in MSIs

A recent scoping review evaluated the available evidence supporting isolation strategies for patients with MSIs in orthopaedic practice (Bessems et al., 2025). Overall, the review did not identify evidence supporting routine ward-level isolation or cohorting of patients with non-MDRO fracture-related infection (FRI) or periprosthetic joint infection (PJI) when standard precautions are consistently applied. In particular, no studies demonstrated a reduction in cross-transmission, surgical site infection, or reinfection rates through the use of dedicated septic wards for non-MDRO MSI patients.

In contrast, the literature consistently supports targeted isolation measures for patients with MDRO, particularly MRSA, vancomycin-resistant *Enterococcus* (VRE), and carbapenemase-producing Enterobacterales (CPE). These recommendations are largely driven by the higher risk of transmission, colonization, and limited therapeutic options associated with MDRO rather than by the diagnosis of MSI itself. Importantly, the review did not identify evidence supporting dedicated septic units. Instead, for selected high-risk pathogens, the available evidence supports single-patient rooms combined with strict adherence to standard and transmission-based precautions.

## 3 Reported practices and perceptions of clinicians

To examine the implementation of isolation policies for MSIs in routine clinical practice and to assess clinicians' perspectives on their necessity, an international survey was distributed to members of the European Bone and Joint Infection Society (EBJIS) during the annual meeting in Bologna, Italy (2025). The survey was designed to explore perceptions regarding room-sharing and isolation practices in clinically relevant MSI scenarios, including the influence of pathogen resistance profiles and wound characteristics.

The questionnaire was distributed to 577 registered EBJIS members, of whom 157 completed the survey in full (response rate: 27.2 %). The full survey instrument is available in the Supplement (Survey S1). Respondents included orthopaedic and trauma surgeons (65.6 %), infectious diseases specialists (24.2 %), microbiologists (5.1 %), infection prevention professionals (2.5 %), plastic surgeons (0.6 %), and pharmacists (0.6 %), representing a broad range of hospital types and levels of experience. Detailed respondent characteristics, including years of professional experience and hospital type, are provided in Table S1 in the Supplement.

Overall, most respondents reported that their institution does not use a dedicated septic ward for the care of patients with MSIs. At the same time, a large majority indicated that isolation policies in their institution differentiate between infections caused by MDRO and those caused by non-MDRO, suggesting broad acceptance of a risk-based distinction at a policy level.

Despite this, individual attitudes towards patient isolation revealed substantial variability, particularly for non-MDRO infections (Table 1). When asked whether a patient with a methicillin-susceptible *S. aureus* (MSSA) PJI without wound complications could share a room with a non-infected orthopaedic patient, more than half (57.1 %) of respondents considered this to be unacceptable. Importantly, responses differed by discipline: orthopaedic surgeons were substantially more likely to oppose room-sharing (70.3 %) than infectious disease specialists (36.8 %) or microbiologists (14.3 %). These findings suggest different risk perceptions between these specialties. However, microbiologist responses should be interpreted cautiously given the small subgroup size ( n=7 ).

**Table 1:** Clinician attitudes towards room-sharing in musculoskeletal infection scenarios.

Clinical scenario	All respondents n/N (%) opposing room sharing	Orthopaedic/trauma surgeons n/N (%) opposing room sharing	ID specialists n/N (%) opposing room sharing	Microbiologists n/N (%) opposing room sharing
MSSA PJI, no wound complications	88/154 (57.1 %)	71/101 (70.3 %)	14/38 (36.8 %)	1/7 (14.3 %)
MSSA FRI, draining fistula	110/151 (72.8 %)	86/100 (86.0 %)	16/36 (44.4 %)	3/7 (42.9 %)
MRSA PJI, no wound complications	128/151 (84.8 %)	86/98 (87.8 %)	29/38 (76.3 %)	7/7 (100 %)

MSSA: methicillin-susceptible *Staphylococcus aureus*; PJI: periprosthetic joint infection; FRI: fracture-related infection; MRSA: methicillin-resistant *Staphylococcus aureus*; ID: infectious diseases.

When the clinical scenario was modified to include a draining fistula in a patient with MSSA FRI, opposition to room sharing increased overall (72.8 %). However, more than half of infectious disease specialists (55.6 %) and microbiologists (57.1 %) still considered isolation to be unnecessary. This indicates that clinicians apply risk stratification based on wound characteristics and perceived local bioburden, although the threshold for isolation differs substantially between disciplines.

In contrast, there was broad agreement regarding infections caused by MDRO. For a patient with MRSA PJI without wound complications, the majority of respondents (84.8 %) considered room sharing to be inappropriate, supporting the use of single-patient rooms for MDRO-associated MSIs. Notably, among orthopaedic and trauma surgeons, the proportion opposing room sharing for a patient with MSSA infection and wound complications was comparable to that for a patient with MRSA infection without wound complications. This finding suggests that, in surgical practice, local wound conditions may be perceived as conferring a transmission risk similar to that associated with MDRO status.

Interestingly, respondents were divided regarding the scientific evidence underpinning isolation strategies. While most agreed that MDRO infections warrant stricter isolation measures, opinions were divided on whether dedicated septic wards are supported by scientific evidence, with 49.2 % of respondents indicating that such evidence exists. Interestingly, this perception was comparable among orthopaedic and trauma surgeons (52.3 %) and infectious disease specialists (48.4 %), whereas microbiologists were markedly less likely to consider septic wards to be evidence-based (14.3 %). This uncertainty persists despite the lack of evidence identified in the scoping review, underscoring a persistent gap between available data and clinical perception.

Across disciplines, however, there was near-universal agreement regarding the importance of standardized care pathways and strict adherence to standard precautions, including hand hygiene, environmental cleaning, and appropriate use of personal protective equipment. This shared consensus provides a common foundation for harmonizing infection prevention practices without relying on extensive structural isolation measures (94.2 %).

## 4 Interpretation and implications for practice

This survey demonstrates a clear gap between available evidence and current perceptions regarding isolation in MSI care. Although most institutions differentiate between MDRO and non-MDRO infections at a policy level, individual attitudes, particularly among orthopaedic and trauma surgeons, remain more restrictive for non-MDRO infections than the literature would support. These findings should be interpreted in the context of a voluntary survey among EBJIS members and may not be fully representative of all clinicians involved in musculoskeletal infection care.

The high clinical burden of MSIs may partly explain this cautious approach. In addition, visible wound characteristics, such as drainage, appear to strongly influence perceived transmission risk. Notably, for many surgeons, MSSA infection with wound complications was viewed as requiring isolation to a similar extent as MRSA infection without wound complications. This finding suggests that visible wound characteristics may influence perceived transmission risk as strongly as pathogen resistance status. Such concerns are biologically plausible given the potential for wound exudate and environmental contamination, although evidence supporting routine isolation on this basis remains limited. While this reflects intuitive risk assessment, evidence supporting increased cross-transmission in such scenarios is lacking. Nevertheless, transmission of *Staphylococcus aureus* between hospitalized patients and via contaminated environmental surfaces has been documented in other healthcare settings. However, the previously mentioned scoping review did not identify evidence demonstrating that these observations translate into a clinically relevant benefit of routine isolation measures in the specific context of non-MDRO musculoskeletal infection care (Bessems et al., 2025).

The finding that nearly half of respondents believe dedicated septic wards are evidence-based further illustrates how historical practices may persist beyond their scientific foundation. Structural separation may provide reassurance, but, in the absence of evidence demonstrating benefit, it must be weighed against its impact on bed capacity, staffing, and patient flow. Room allocation decisions may also be influenced by factors other than transmission prevention. Patients with musculoskeletal infections often require complex wound care, prolonged antimicrobial treatment, and repeated clinical assessments, for which single-room placement may be preferred for reasons of privacy, comfort, or operational considerations independent of infection control.

Importantly, there was near-universal agreement on the value of standardized care pathways and strict adherence to standard precautions. This shared consensus provides a practical way forward. However, any risk-based approach is dependent on consistent implementation of infection prevention measures, including hand hygiene, environmental cleaning, and appropriate use of personal protective equipment, for which compliance may vary across institutions (Erasmus et al., 2010).

Taken together, the available evidence supports a differentiated, risk-based approach: routine ward-level isolation for non-MDRO MSIs cannot be justified when standard precautions are consistently applied, whereas infections caused by MDRO (particularly MRSA) may warrant single-patient rooms and transmission-based precautions, depending on the specific organism and local risk assessment. Such an approach enables infection prevention measures to be directed where they are most likely to provide benefit while maintaining high standards of patient care.

## 5 Conclusion

Isolation practices in MSI care appear to be shaped as much by historical precedent and perceived risk as by scientific evidence. While targeted isolation for MDRO, particularly MRSA, remains justified, routine ward-level separation of non-MDRO infections is currently not supported by available evidence. Our survey demonstrates that this distinction is not consistently reflected in clinical attitudes, especially when visible wound characteristics influence perceived transmission risk.

Moving forward, orthopaedic infection care should transition from tradition-driven structural segregation toward a differentiated, risk-based strategy grounded in evidence and reinforced by rigorous adherence to standard precautions. Sustained investment in training, education, and auditing to ensure high compliance with these precautions may help bridge the gap between long-standing practice patterns and evolving evidence. Such alignment is essential not only to ensure scientific integrity but also to optimize resource use and maintain high-quality, sustainable care in modern healthcare systems.

## Supplement

10.5194/jbji-11-489-2026-supplement The supplement related to this article is available online at https://doi.org/10.5194/jbji-11-489-2026-supplement.

## Data Availability

The data supporting the findings of this study are presented in the paper and Supplement.
