# Supplementary material for: Isolation policies in musculoskeletal infection care: time to move from tradition to evidence
Source: J Bone Jt Infect. 2026 Aug 7;11(4):489–92. doi: 10.5194/jbji-11-489-2026 (PMC13479932; doi:10.5194/jbji-11-489-2026)
Supplement: Supplementary file 1 [file jbji-11-489-2026-supplement.pdf]

Supplement of J. Bone Joint Infect., 11, 489–492, 2026
https://doi.org/10.5194/jbji-11-489-2026-supplement
© Author(s) 2026. CC BY 4.0 License.

Supplement of

Isolation policies in musculoskeletal infection care: time to move from tradition to evidence

Laura Bessems et al.

Correspondence to: Willem-Jan Metsemakers (willem-jan.metsemakers@uzleuven.be)

The copyright of individual parts of the supplement might differ from the article licence.

Survey S1. Full questionnaire distributed to members of the European Bone and Joint Infection Society (EBJIS) during the 2025 Annual Meeting.

QUESTIONNAIRE ISOLATION POLICY

Section A: Current Isolation Practices in Your Center

In which country are you currently employed? ______________________________

What is your current speciality or professional role?

☐ Orthopaedic/Trauma surgeon
☐ Plastic surgeon
☐ Infectious disease specialist
☐ Microbiologist
☐ Pharmacist
☐ Epidemiologist
☐ Infection control and prevention
☐ Other: ______________________________

How many years of experience do you have in your current field?

☐ Student/Resident
☐ < 5 years
☐ 5–10 years
☐ 11–20 years
☐ > 20 years

What is the size or type of your hospital?

☐ Small hospitals: fewer than 100 beds
☐ Medium hospitals: 100 to 499 beds
☐ Large hospitals: 500 or more beds
☐ University hospitals: 500 or more beds

Does your center currently use a dedicated septic ward (separate unit) for musculoskeletal infection cases?

☐ Yes ☐ No ☐ No answer

Does your surgical ward currently have separate rooms available to isolate a single patient with a musculoskeletal infection (regardless of resistance pattern)?

☐ Yes ☐ No ☐ No answer

Do you currently differentiate isolation policies for musculoskeletal infection patients based on whether their pathogens are multidrug-resistant (MDR) or not?

☐ Yes ☐ No ☐ No answer

Does your center have a clearly defined standardized care package for musculoskeletal infection that includes standard precautions (e.g. hand hygiene, use of protective equipment, cleaning protocols)?

☐ Yes ☐ No ☐ No answer

Section B: Case scenarios

A patient is scheduled for elective total hip arthroplasty.

The adjacent bed is occupied by an individual diagnosed with an acute Methicillin-susceptible *Staphylococcus aureus* periprosthetic joint infection who does not have wound-related complications. Is it acceptable to place these two patients in the same room?

☐ Yes
☐ No
☐ No answer

The adjacent bed is occupied by someone with an Methicillin-susceptible *Staphylococcus aureus* fracture-related infection with a draining fistula. Is it acceptable to place these two patients in the same room?

☐ Yes
☐ No
☐ No answer

The adjacent bed is occupied by an individual diagnosed with an Methicillin-resistant *Staphylococcus aureus* periprosthetic joint infection who does not have wound-related complications. Is it acceptable to place these two patients in the same room?

☐ Yes
☐ No
☐ No answer

Section C: Personal view on Isolation Practices

Is there scientific evidence to support the implementation of dedicated septic wards for musculoskeletal infection patients?

☐ Yes ☐ No ☐ No answer

Is there scientific evidence to support the need for stricter isolation measures for musculoskeletal patients with multidrug-resistant pathogens compared to those with non-multidrug-resistant pathogens?

☐ Yes ☐ No ☐ No answer

Do you agree that it is important to have a clearly defined standardised care package (e.g. hand hygiene, use of protective equipment, cleaning protocols) implemented for the prevention of musculoskeletal infection patients?

☐ Strongly Agree
☐ Agree
☐ Neither agree nor disagree
☐ Disagree
☐ Strongly Disagree
☐ Not sure/not applicable
☐ No answer

Thank you for taking your time to participate in this survey

We value your input and feedback.
